# Self-pacing ameliorates recall deficit when listening to vocoded discourse: a cochlear implant simulation

**DOI:** 10.3389/fpsyg.2023.1225752

**Published:** 2023-11-20

**Authors:** Thomas A. Hansen, Ryan M. O’Leary, Mario A. Svirsky, Arthur Wingfield

**Affiliations:** ^1^ Department of Psychology, Brandeis University, Waltham, MA, United States; ^2^ Department of Otolaryngology, NYU Langone Medical Center, New York, NY, United States

**Keywords:** discourse memory, noise-band vocoding, self-paced listening, linguistic context, semantic hierarchy effect

## Abstract

**Introduction:**

In spite of its apparent ease, comprehension of spoken discourse represents a complex linguistic and cognitive operation. The difficulty of such an operation can increase when the speech is degraded, as is the case with cochlear implant users. However, the additional challenges imposed by degraded speech may be mitigated to some extent by the linguistic context and pace of presentation.

**Methods:**

An experiment is reported in which young adults with age-normal hearing recalled discourse passages heard with clear speech or with noise-band vocoding used to simulate the sound of speech produced by a cochlear implant. Passages were varied in inter-word predictability and presented either without interruption or in a self-pacing format that allowed the listener to control the rate at which the information was delivered.

**Results:**

Results showed that discourse heard with clear speech was better recalled than discourse heard with vocoded speech, discourse with a higher average inter-word predictability was better recalled than discourse with a lower average inter-word predictability, and self-paced passages were recalled better than those heard without interruption. Of special interest was the semantic hierarchy effect: the tendency for listeners to show better recall for main ideas than mid-level information or detail from a passage as an index of listeners’ ability to understand the meaning of a passage. The data revealed a significant effect of inter-word predictability, in that passages with lower predictability had an attenuated semantic hierarchy effect relative to higher-predictability passages.

**Discussion:**

Results are discussed in terms of broadening cochlear implant outcome measures beyond current clinical measures that focus on single-word and sentence repetition.

## Introduction

Spoken discourse is ordinarily understood and encoded into memory with ease. However, the apparent ease of this process can obscure the number and complexity of the underlying cognitive operations that lead to successful comprehension. These operations include: (1) extracting acoustic patterns from the transient signal and encoding phonology, (2) matching the input phonology with representations in the mental lexicon to identify individual words, (3) determining the syntactic and semantic relationships between words as they form sentences, and finally, (4) integrating the semantic output of these sentences to comprehend the overall communication (van Dijk and Kintsch, 1983; Haberlandt and Graesser, 1989; Gernsbacher, 1990). It can further be assumed that many of these operations, and their subprocesses, are both interactive and overlapping in time.

Those operations that cannot be conducted online, as the speech is arriving, must be conducted on a transient, capacity-limited, memory trace of the preceding input (Jarvella, 1971; Fallon et al., 2004), often characterized in terms of working memory resources (Kintsch, 1974; Carpenter et al., 1994; Daneman and Merikle, 1996; Wingfield, 2016). Finally, for complete success, the listener must be able to recall the content of what has been heard. In this latter regard, it has long been recognized (e.g., Bartlett, 1932) that the line between comprehension and recall accuracy is not a sharp one, such that the better one understands the meaning of a communication the more likely it will be accurately recalled (Schank and Abelson, 1976; Thorndyke, 1977).

The challenge of processing spoken discourse becomes significantly more pronounced under difficult listening conditions. These could be external factors, such as the presence of background noise, or internal ones, such individuals dealing with decreased hearing acuity. Under such circumstances, lexical identification and its underlying subprocesses might become slower and more susceptible to errors.

There is a special case of perceptual challenge that has attracted increasing attention in the speech comprehension literature. This is the case of adults whose hearing loss has progressed beyond the benefit from conventional hearing aids and who have received cochlear implants to recover functional hearing abilities (Svirsky, 2017).

Unlike conventional hearing aids, that amplify natural speech, cochlear implants are surgically implanted devices that electrically stimulate the auditory nerve directly via tonotopically arrayed electrodes placed within the cochlea. Often thus referred to as “electrical hearing,” cochlear implants are the most widely employed sensory neuroprostheses in regular use. As of December 2019, approximately 118,100 adults and 65,000 children in the United States were users of cochlear implants^1^, with an increasing number of older adults aged 70 and older receiving cochlear implants (Lin et al., 2012).

While implants may have up to 22 intra-cochlear electrodes, factors such as current spread, tonotopic mismatch, and neural survival, may limit the spectral resolution of the acoustic information available to the listener. These and other factors can often result in cochlear implant users receiving speech with the equivalent of only 4–8 frequency channels (Friesen et al., 2001; Fu and Nogaki, 2005; Perreau et al., 2010; see also Faulkner et al., 2001). Although, the possibility has been raised that modern cochlear implants may grant slightly higher spectral resolution than is often reported (Croghan et al., 2017). Nonetheless, the result is speech that is sharply degraded as compared to normal speech.

A potential counter to this challenge is the facilitation offered by a constraining linguistic context that can increase the probability of a target word, hence lowering the recognition threshold for that word (e.g., Amichetti et al., 2018). In everyday discourse early estimates suggested that the mean probability of words in everyday speech may range from 0.3 to 0.5 based on context supplied by syntactic and semantic constraints (Chapanis, 1954; Shannon, 1951).

There are a number of well-developed models that focus on phonological and contextual factors in word recognition (cf., Morton, 1969; McClelland and Elman, 1986; Marslen-Wilson and Zwitserlood, 1989; Luce and Pisoni, 1998). One of the few attempts to offer a fully encompassing framework for speech comprehension, from word recognition through to the understanding of full discourse, for clear or degraded speech, can be found in the ELU (*Ease of Language Understanding*) model developed by Rönnberg et al. (2013). Similar to more limited models, the ELU model has thus far focused on quantitative effects of a degraded input, while giving less attention to potential qualitative effects on discourse comprehension and recall. In the present study, we investigate qualitative effects on speech comprehension that, as we will suggest, should be incorporated into models of speech comprehension such as the ELU model.

We had two major hypotheses when conducting this experiment. First, we hypothesized that the effect of acoustic degradation on comprehension of spoken discourse would lead not only to quantitative deficits in recall performance, but that there would also be a qualitative effect. This, qualitative effect, we suggest, would be reflected in the hierarchical structure of propositions that participants recalled. Our second hypothesis was that recall of degraded speech can be improved if listeners are allowed to control the rate of presentation of the speech input.

### Simulating the sound of a cochlear implant

In the experiment to be described, young adults with age-appropriate hearing were asked to recall recorded speech passages. The passages had either high or low average inter-word predictability derived from published norms (Miller and Coleman, 1967; Acquino, 1969) and were presented with clear speech or with noise-band vocoding to simulate speech as heard via a cochlear implant.

As will be described in greater detail in the Methods section, noise-band vocoding is a sound processing algorithm that separates natural speech into a specified number of frequency bands with the extracted amplitude profile of each band used to modulate noise whose frequency range is the same as that of the corresponding analysis filter. When the outputs of all frequency bands are recombined, the result can be perceived as speech; the more frequency bands (channels) the more natural the resulting speech will sound. Conversely, reproduction with fewer frequency bands (e.g., 6 channel vocoding) results in the unnatural, sharply distorted quality that approximates the speech signal available to many CI users (Shannon et al., 1995).

It should be noted that vocoded speech, conventionally expressed in terms of the number of frequency channels available to the listener, is not an exact replication of the sound produced by a cochlear implant due to factors such as perceptual “smearing” and frequency mismatch as may occur for post-lingually deaf adults after implantation (Svirsky et al., 2021). Vocoding has nevertheless received wide use in simulation studies as an approximation to implant hearing that allows tight control of the spectral information available to the listener (Everhardt et al., 2020).

### Qualitative versus quantitative effects on recall

Based on prior literature, one would expect better recall for passages with higher relative to lower inter-word predictability (Acquino, 1969), and better recall for stimuli presented in clear speech relative to vocoded speech (Ward et al., 2016). However, our primary interest is whether these factors may also lead to qualitative differences in recall. For this purpose, we took advantage of a formalized representational system that organizes discourse elements into a hierarchical array that indexes the relative importance of different elements to the overall understanding of a discourse passage (Kintsch and van Dijk, 1978; Meyer, 1985; Kintsch, 1988). Consistent with this formulation, one often sees a semantic hierarchy effect (in the discourse literature this is often called a “levels effect”; Kintsch and van Dijk, 1978), in which information that is higher in the hierarchy (the main ideas represented in a passage) are better remembered than information lower on the hierarchy (details that embellish or add subsidiary specifics to the main ideas).

While the semantic hierarchy effect may be moderated by recall of details salient to a particular individual (Anderson and Pichert, 1978; Mandel and Johnson, 1984), an overall semantic hierarchy effect in discourse recall has been reliably observed for both written and spoken discourse (e.g., Dixon et al., 1984; Mandel and Johnson, 1984; Zelinski et al., 1984; Stine and Wingfield, 1987). The appearance of a semantic hierarchy effect is thus typically taken as evidence that a listener (or reader) has developed an understanding of the text sufficiently to discriminate among main ideas, mid-level details and lower-level details represented in the discourse (Kintsch and van Dijk, 1978; van Dijk and Kintsch, 1983).

If a reduction in either inter-word predictability or reduced spectral clarity slows successful word recognition and/or draws resources that would otherwise be available for developing an understanding of the discourse structure, one would expect a detrimental effect on overall comprehension of the discourse passage. To the extant this is the case, this would result in quantitatively poorer passage recall. Qualitatively, one would expect to see a weakening or attenuation of the semantic hierarchy effect in these conditions. Such an attenuation would appear in the form of an absence, or reduction, in the difference between recall of main ideas versus lower-level details. This prediction is grounded in past observations that the semantic hierarchy effect is reduced for passages with lower inter-word predictability and in cases of increased task difficulty, such as when the speech rate is increased (Titone et al., 2000). An attenuation of the semantic hierarchy effect has also been shown in patients with right hemisphere brain lesions, who are known to have difficulty understanding the gist of discourse (Titone et al., 2001).

### Self-regulation of input rate

If it is the case that degrading speech slows word recognition (Miller and Wingfield, 2010; Cousins et al., 2014), with effects that cascade to slow comprehension of the full discourse, one would predict that allowing listeners to control the rate of speech input would ameliorate the effects of vocoding on recall. Support for this possibility can be seen in a study by Piquado et al. (2012) who compared discourse recall by young adults with age-normal hearing and participants of similar age, years of formal education, and vocabulary, who had mild to moderate hearing loss. The two participant groups heard recorded passages either without interruption or with intermittent interruptions where the listener was allowed to control the initiation of the next segment of the speech.

This technique, sometimes referred to as an “auditory moving window” technique (Ferreira et al., 1996), or “self- paced listening” (Fallon et al., 2006), allowed the listeners to pace themselves through recorded passages on a segment-by-segment basis, initiating each subsequent segment with a keypress when they felt ready to hear the next segment. When presented without interruption, the hearing-impaired listeners’ recall was poorer than that of the normal-hearing participants. However, when allowed to self-pace the speech input, the hearing-impaired group not only showed a significant improvement in recall, but in this particular case, actually matched the recall accuracy of the normal-hearing participants (Piquado et al., 2012).

Although the study by Piquado and colleagues is suggestive, it cannot automatically be assumed that self-pacing will rescue the presumed detrimental effects of vocoding on passage recall. This is primarily due to the spectrally impoverished character of vocoded speech (and speech heard via a cochlear implant) that significantly deviates from the sound of regular speech. We address this question in the present study by comparing passage recall for clear and vocoded speech presented with continuous and self-pacing formats.

## Methods

### Participants

The participants were 24 university students and staff (16 men, 7 women, and one non-binary) with ages ranging from 18 to 26 (*M* = 20.5 years, *SD* = 1.96). Because this was a listening task, audiometric evaluation was carried out for each participant using a Grason-Stadler AudioStar Pro clinical audiometer (Grason-Stadler, Inc., Madison, WI) using standard audiometric techniques in a sound-attenuating testing room. The participants had a mean better-ear pure tone average (PTA) based on thresholds averaged across 0.5, 1, 2, and 4 kHz of 6.88 dB HL (*SD* = 4.12), placing them in the range of normal hearing sensitivity (Katz, 2002).

#### Vocabulary knowledge

All participants received a 20-item version of the Shipley vocabulary test (Zachary, 1991). The Shipley is a written multiple-choice test in which the participant is asked to indicate which of six listed words has the same or nearly the same meaning as a given target word. The group’s mean score was 13.00 (*SD* = 1.80) out of 20 possible points.

#### Working memory

Working memory was assessed using an adapted version of the reading span (R-span) task (Daneman and Carpenter, 1980; Stine and Hindman, 1994). In this task participants were presented with sets of sentences, ranging from a single sentence up to five consecutive sentences. After each sentence was presented, participants were asked whether it was true or false. After all the sentences in a set had been presented, the participants were asked to recall the last word of each sentence in the order in which they were presented within the set. Hence, the reading span task captures the dual aspect of working memory, requiring concurrent storage and processing of information in immediate memory (Postle, 2006; McCabe et al., 2010).

The scoring procedure used for the R-Span followed McCabe et al.’ (2010) procedure in which participants received three trials for each set-size of sentences. Regardless of accuracy, participants received all three trials for sets up to three sentences, after which the task was ended when a participant failed to recall any of the sentence-final words within a given set. The working memory score was taken as the total number of trials in which all sentence-final words were recalled correctly in the correct order of their presentation in a sentence set. The group’s mean R-Span score was 9.42 (*SD* = 2.98) out of a possible total of 15. These vocabulary and R-Span scores are within the range often found for university undergraduates and were collected in an exploratory way as possible predictors of condition-effect performance.

### Stimuli

The stimulus materials consisted of eight prose passages approximately 150 words in length that covered a variety of topics, such as instructions on to how to make a kite, information about the Oceanarium in Florida, or a story about a conversation between a nobleman and a merchant who met in a tavern. Each of the passages had been previously normed for mean inter-word predictability using a “cloze” procedure (Miller and Coleman, 1967). These norms were based on the percentage of individuals who give a particular word as the most likely missing word when a word is deleted from the passage. Cloze probabilities serve as a convenient summary statistic that reflects the combined effects of the syntactic, semantic, and pragmatic constraints that operate on word choice. Prior work using these passages has shown that their cloze predictability values correlate highly with subjective estimates of passage difficulty as well as actual passage recall (Acquino, 1969; Riggs et al., 1993). Four of the eight passages, which we refer to as *low-predictability passages,* had a mean inter-word predictability rating of 0.51, and four passages, referred to as *high-predictability passages,* had a mean inter-word predictability rating of 0.67.

Each of the passages was recorded by a female speaker of American English at an average speech rate of 150 words per minute (wpm) onto computer sound files using Sound Studio v2.2.4 (Felt Tip, Inc., New York, NY) that digitized (16-bit) at a sampling rate of 44.1 kHz. Recordings were equalized within and across passages for root-mean-square (RMS) intensity using Praat (Boersma and Weenink, 2022). For the self-paced listening condition, the original recordings were re-recorded with markers placed at major clause and sentence boundaries that would signal the presentation computer to interrupt the passage at these points. The presentation computer was programmed to initiate each subsequent segment with a participant’s keypress, and to record the elapsed time between the interruption of the input and the participant’s keypress to initiate the next segment. The mean number of segments per passage for the high and low predictability passages was 10.50 and 10.62 segments, respectively. The mean number of words per segment for the high and low predictability passages was 7.91 and 7.97 words, respectively.

#### Vocoding

Each of the passages that had been prepared for continuous or self- paced presentation were processed using 6-channel noise-band vocoding, following the method outlined by Shannon et al. (1995) and implemented using MATLAB (MathWorks, Natick, MA). Broadband speech (80–8,000 Hz) was band-pass filtered (3rd order Butterworth filter with 18db/octave rolloff) into logarithmically spaced frequency bands, with the amplitude envelope of each frequency band extracted and then low pass filtered with a 300 Hz cutoff frequency. The amplitude envelope extracted from each frequency band was then used to modulate white noise which had been filtered by the same band-pass filter that isolated the frequency band of that envelope. The resultant signals from all the bands were then recombined to produce the vocoded stimuli. The vocoded stimuli were matched for RMS amplitude with the unprocessed stimuli (Kong et al., 2015).

### Procedure

A within-participants design was used in which each participant heard all eight passages, with four passages presented in a continuous format and four presented in the self-pacing format. Of these, two of the passages in the continuous and self-pacing formats were heard with clear (non-vocoded) speech, and two were heard with 6-channel vocoding. Within each set of two passages heard in each pacing format and speech clarity condition, one was high and the other was low predictability. The particular passages heard in each condition were counterbalanced across participants such that, by the end of the experiment, each passage had been heard an equal number of times in its continuous or self-pacing format, and in clear speech or with 6-channel vocoding.

Participants were told they would hear a series of recorded passages; some would be presented clearly, and some would be heard with an acoustic distortion. In either case their task was to listen to the passage and attempt to recall as much as they could remember from the passage as accurately and completely as they could when the passage had ended. They were further told that some passages would be presented in a normal manner without interruption, while others would be presented in a way that allowed them to pace through the passage at their own rate. They were told that, for these passages, the passage would be halted from time-to-time. When a passage stopped, they could initiate the next segment when they felt they were ready for it by pressing an indicated key on the computer keyboard. They were told they could pause before initiating the next segment for as long or short of a time as they wished, but that their goal was to be able to recall as much of the passage as possible after it was finished.

Participants were told before each passage whether it would be continuous or presented for self-pacing, and whether it would be clear or distorted. No mention was made that in the self- paced condition that the computer would also be recording the duration of their pause between the end of each segment and their keypress to initiate the next segment. Similarly, no mention was made that some passages had high or low inter-word predictability. Stimuli were presented binaurally via Eartone 3A insert earphones (E-A-R Auditory Systems, Aero Company, Indianapolis) at 65 dB HL.

After the passage ended, a question mark was presented on the screen indicating that the participant could begin their free recall. Participants had as much time as they needed to be able to recall as much of the passage as possible, as accurately as they could. When they felt that they had recalled as much of the passage as possible, they could initiate the next trial to hear the following passage.

Participants received a two-part familiarization session prior to beginning the main experiment. First, participants were introduced to the self-pacing format, in which a passage in clear speech was presented with the self-pacing recall instructions, and sound level as would be used in the main experiment. On completion of their self-paced presentation and recall, participants were asked if they had any questions regarding the experimental task. This passage was not used in the main experiment.

To familiarize the participants with the sound of vocoded speech, participants were presented with a 10-min recorded podcast processed with 6-channel vocoding. Participants were allowed to read along with a written transcript as they were hearing the vocoded speech to aid in their adaptation (Erb et al., 2012).

Written informed consent was obtained from all participants following a protocol approved by the Brandeis University Institutional Review Board.

### Scoring for recall accuracy

Recall performance was assessed using the propositional framework described by van Dijk and Kintsch (1983) and Turner and Greene (1978). In this analysis, propositions are defined as words such as verbs and adjectives that have significance in relation to a content word (nouns) that comprise the ‘argument’ of the proposition. Propositions can also take other propositions as their arguments. This nesting of propositions allows for a hierarchical arrangement of the propositions within a discourse passage.

Based on this hierarchical structure, the propositions within each of the eight passages were divided into three levels: *main ideas*, defined as propositions whose arguments were directly related to the overall meaning of the passage, *mid-leve*l propositions were those that took main propositions as their arguments, and *details,* that were propositions that took mid-level or other minor propositions as their arguments.

Individual propositions were identified using the Computerized Propositional Idea Density Rater (CPIDR) 5.1 software (Brown et al., 2008; Covington, 2012) following the procedures outlined in Turner and Greene (1978). Propositions were scored as correct or incorrect, with no half-credits given. Close synonyms that did not affect the meaning of a proposition received full credit.

### Data analysis

Recall data were analyzed using linear mixed effects models with participants and items set as random intercepts with the slope of the semantic hierarchy effect allowed to vary by items [LMEM’s, lme4 package version 1.1–19 (Bates et al., 2015), lmerTest package version 3.1–3 (Kuznetsova et al., 2015) in R-Studio (RStudio Team, 2020)]. A reverse selection approach was used to select a final model including only significant predictors and interactions. After determining the final model through model comparisons, null models for each of the predictors within the final model were created. All reported *p*-values, unless stated otherwise, were obtained through likelihood-ratio tests performed by comparing a null model for each predictor to the final model.

## Results

**Figure 1:**
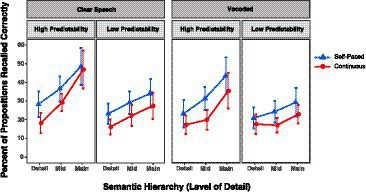
Percentage of details, mid-level information, and main ideas recalled from passages when presented in an uninterrupted of self-paced format for clear speech (left panels) and vocoded speech (right panels). Error bars are one standard error.

Figure 1 shows the percentage of propositions representing details, mid-level information, and main ideas correctly recalled by participants when the passages were presented with a continuous or self-paced format. Data are shown for high and low predictability passages when heard with clear (non-vocoded) speech (left panel) and 6-channel vocoded speech (right panel).

The data shown in Figure 1 were analyzed using a linear mixed effect model with the final model containing Semantic Hierarchy (details, mid-level information, main ideas), Clarity (vocoded, clear speech), Format (continuous, self-paced), and passage Predictability (high, low) as fixed effects, with Participants and Items (passages) as random intercepts. The outcome is given in Table 1.

**Table 1:** Overall linear mixed effects model for passage recall.

Predictor	*𝜒* ^2a^	df^b^	*p* ^c^
Semantic hierarchy	19.93	4	**< 0.001**
Clarity (natural vs. vocoded)	10.41	1	**0.002**
Format (continuous vs. self-pacing)	17.90	1	**< 0.001**
Predictability (high vs. low)	10.06	3	**0.018**
Semantic hierarchy X predictability	8.02	2	**0.018**
Semantic hierarchy X format	1.43	2	0.490
Semantic hierarchy X clarity	2.68	2	0.262
Format X predictability	0.50	1	0.481
Format X clarity	0.02	1	0.880

^a^𝜒^2^ value for comparisons of each step of the model.

^b^Degrees of freedom for the 𝜒^2^ test.

^c^Value of *p* reflects significance of change in model fit at each step of the model.

Significant values of *p* are indicated in bold.

As would be expected, recall accuracy was on average 7.85 percentage points greater for high predictability passages than low predictability passages (*p* = 0.018). There was also a significant semantic hierarchy effect, in which recall was on average 9.78% better for main ideas relative to mid-level information, and on average 5.61% better for mid-level information relative to minor details (*p* < 0.001). Also confirmed was a main effect of presentation format, in which recall was on average 6.83% superior when listeners were allowed to control the presentation rate by self-pacing, relative to recall accuracy when passages were presented without interruption (*p* < 0.001). In addition, a significant effect of clarity was confirmed in the form of 4.78% better recall for passages heard in clear speech than passages presented with 6-channel vocoding (*p* = 0.002). A significant Semantic Hierarchy effect X Predictability interaction (*p* = 0.018) reflected a weaker semantic hierarchy effect, seen as a shallower slope for the low predictability passages relative to the high predictability passages.

The recall benefit of self-pacing relative to a continuous presentation appeared across experimental conditions, an observation consistent with the absence of Format X Predictability (*p* = 0.481), Format X Clarity (*p* = 0.880), or Format X Semantic Hierarchy (*p* = 0.490) interactions. Visual inspection of the extreme right panel of Figure 1 might suggest that the combination of low predictability, vocoding, and a continuous presentation format resulted in the shallowest semantic hierarchy effect. This was not, however, supported by a significant Semantic Hierarchy X Clarity interaction (*p* = 0.262) or four-way interaction of Semantic Hierarchy X Clarity X Format X Predictability (*p* = 0.684). It is also possible that the suggestion of a differentially shallower slope of the semantic hierarchy effect for the low predictability vocoded passages presented in the continuous format was due to a functional floor for recall of details (none of the remaining three-way interactions were significant).

### Self-pacing pause durations

The mean duration of participants’ inter-segment pauses was 2.43 s (*SD* = 2.71 s), indicative of a wide variability that appeared both between and within participants. In the former case, we found that those participants who tended on average to pause for longer periods between segments showed better recall than those who tended to pause for shorter periods, confirmed by a significant effect in a linear regression *F* (1, 22) = 10.12, *p* = 0.004. Within this overall between-participant difference, we found that pausing longer in self-pacing a passage was associated with significantly better recall on that passage, 𝜒^2^ (1) = 12.83, *p* < 0.001. These general patterns did not differ among experimental conditions.

Pause durations were not affected by individual differences in hearing acuity (*p* = 0.335), Shipley Vocabulary score (*p* = 0.945), or R-Span score (*p* = 0.394).

### Working memory and recall performance

Working memory capacity as indexed by R-Span scores was a significant predictor of overall recall performance, 𝜒^2^ (1) = 5.855, *p* = 0.016. Mixed-effects modeling did not reveal any significant interactions between R-Span score and the size of the semantic hierarchy effect (*p* = 0.156), stimulus clarity (*p* = 0.549), passage predictability (*p* = 0.355), or presentation format (*p* = 0.769). There was no significant effect of R-Span scores on the size of the self-pacing benefit, F (1, 22) = 0.04, *p* = 0.851.

For this participant sample, neither vocabulary score, 𝜒^2^ (1) = 1.31, *p* = 0.253, nor hearing acuity, 𝜒^2^ (1) = 0.76, *p* = 0.385, affected recall accuracy.

## Discussion

A striking feature of spoken language is the rapidity of the input, with conversational speech rates ranging between 140 to 180 words per minute (wpm), as “slow” as 90 wpm in thoughtful conversation, and well over 200 wpm for a radio or TV newsreader working from a prepared script (Stine et al., 1990). It is thus not surprising that adults who hear via cochlear implants have a special difficulty when confronted by rapid speech (Ji et al., 2013; Winn and Teece, 2021; O’Leary et al., 2023), where natural limits on the rate at which speech input can be perceptually processed and encoded in memory are compounded by the sharply degraded signal produced by the implant.

In the present experiment we presented normal-hearing young adults with 6-channel noise-band vocoded speech to simulate the spectrally limited speech as delivered by a cochlear implant (Shannon et al., 1995; Everhardt et al., 2020). As would be expected (Acquino, 1969; Riggs et al., 1993), overall recall of discourse passages with higher average inter-word predictability was superior to recall of passages with lower inter-word predictability. As also would have been expected, in both cases, noise-band vocoding significantly depressed recall for the same passages, relative to when they were heard with clear speech. Our primary interest, however, is whether inter-word predictability and/or noise-band vocoding would produce a qualitative effect on recall. For this purpose, we focused on the effects of these variables on the presence and size of the semantic hierarchy effect in participants’ recall.

### Effects of conditions on the semantic hierarchy effect

As previously noted, the semantic hierarchy effect describes the reliable finding that participants’ recall of meaningful spoken or written text tends to show better recall for main ideas than mid-level information or details from a discourse passage (Meyer, 1985; Stine and Wingfield, 1987). This observed pattern has been interpreted in the discourse processing literature as evidence of the individual’s understanding of the overall meaning of a passage (e.g., Kintsch, 1974; Thorndyke, 1977; Kintsch and van Dijk, 1978; van Dijk and Kintsch, 1983). To these theorists, it is the ability to determine which elements are the main ideas in the discourse and which are potentially less central details that underlies a successful memory representation. The appearance of a significant semantic hierarchy effect under all conditions in this experiment thus suggests that even with low predictability passages and vocoded speech, participants’ memory representations reflected the passage structure.

Within this overall finding, several significant effects and trends were observed. Most notably, passages with lower inter-word predictability had a shallower slope to their semantic hierarchy effect relative to the high inter-word predictability passages. This attenuation of the size of the semantic hierarchy effect was also associated with poorer passage recall. It would be consistent with our current framework to see this as a causal relationship, although this cannot be confirmed with the present data.

A strong impact of self-pacing was evident, which significantly enhanced recall for both clear and distorted speech. However, this effect was additive rather than multiplicative, with the influence of self-pacing being relatively similar across both predictability and clarity conditions. Additionally, self-pacing did not significantly alter the size of the semantic hierarchy effect, as reflected in the roughly similar slopes in the continuous and the self-paced conditions.

To the extent that the size of the semantic hierarchy effect is indicative of the listener’s understanding of a discourse passage, one might have expected the smallest semantic hierarchy effect (the shallowest slope) to appear for the low predictability, vocoded passages, presented in the continuous format. While this trend was observed, it did not reach statistical significance, thus leaving passage predictability as the major determiner of the size of the semantic hierarchy effect in recall.

A second unmet expectation was that participants might pause for longer periods when confronted by vocoded speech than clear speech to give themselves more time to process the degraded input. Contrary to this expectation, pause durations appeared insensitive to a difference in stimulus clarity. This finding, however, is consistent with an observation reported in the previously cited study by Piquado et al. (2012). These authors also found that self-pacing pause times were not sensitive to speech clarity and mean pause durations did not differ for participants with hearing loss versus those with normal hearing.

Although pause times were not sensitive to stimulus clarity, it was the case that participants tended to perform better when they adaptively paused for longer durations between segments. We cannot, however, say with the present data what distinguished those participants who tended to show overall longer pause times on average from those who tended to pause for shorter durations. It may be that those who generally pause for longer or shorter periods is reflective of the individual’s meta-awareness of their memory capacity, or their ability to monitor the cumulative memory load as more information arrives (e.g., Nelson, 1990; Amichetti et al., 2013). One may speculate that personality factors such as differences in self-efficacy and control beliefs (Bandura, 1997; Riggs et al., 1997), or cognitive factors such as differences in efficiency of memory updating as a component of executive function (McCabe et al., 2010), may be contributors to pause duration decisions. We suggest this as an area for future research.

Working memory plays a central role in the previously cited ELU model (Rönnberg et al., 2013), where it is seen as encompassing a number of cognitive functions relevant to language understanding. An essential feature of working memory is the postulate of a trade-off between processing and storage, whether characterized in terms of a shared general resource (Just et al., 1982; Carpenter et al., 1994), or a limited-capacity central executive (Baddeley, 2012).

However, conceived, one of our goals was to determine whether individual differences in working memory capacity influence task performance, and if so, what operations may specifically be affected. As we found, working memory capacity as indexed by the commonly used R-Span task did affect passage recall, and it did so regardless of experimental condition. On the other hand, the degree of benefit of self-pacing on recall, relative to passages presented in a continuous format, was not influenced to a significant degree by individuals’ working memory capacity. It is possible that a more sensitive measure of working memory or a measure of individual differences in executive control may have served as a better predictor of the degree of self-pacing benefit.

## Conclusion

Allowing a listener to meter the flow of a discourse passage results in better recall of the discourse content, a finding that holds for passages with high and low inter-word predictability, and for passages presented with clear speech or with a vocoder simulation of the spectrally limited quality of speech when delivered via a cochlear implant.

One may draw a rough parallel between the self-pacing paradigm employed here and the way in which listeners control the temporal pacing of speech from a communication partner. For example, studies of turn-taking in everyday conversation have shown that individuals subtly, through spontaneous use of eye contact, or overtly, by a verbal request, encourage a speaker to pause occasionally to aid comprehension. Or, in a case closer to our present paradigm, if the speaker has already paused, when to resume speaking (e.g., Wilson et al., 1984; Dequtyte and Astell, 2021).

Current clinical tests to measure the efficacy of cochlear implants, as well as conventional hearing aids, primarily evaluate the ability to repeat standardized, isolated words (Peterson and Lehiste, 1962) and short sentences (Spahr et al., 2012). This present vocoder simulation experiment suggests the importance of extending outcome measures, not only to include recall of discourse-length materials, but also the extent to which an implant user can take an active role in guiding a speaker’s use of pauses to allow the implant user additional time to process what has been heard. As this experiment has demonstrated using a vocoder simulation of cochlear implant hearing, there is significant value to self-directed control of input rate for effective recall of what has been heard.

## Data availability statement

The raw data supporting the conclusions of this article will be made available by the authors, without undue reservation.

## Ethics statement

The studies involving humans were approved by the Brandeis Institutional Review Board. The studies were conducted in accordance with the local legislation and institutional requirements. The participants provided their written informed consent to participate in this study.

## Author contributions

All authors listed have made a substantial, direct, and intellectual contribution to the work and approved it for publication.

## Footnotes

^1^ https://www.nidcd.nih.gov/health/cochlear-implants
